# MegaTevs: single-chain dual nucleases for efficient gene disruption

**DOI:** 10.1093/nar/gku573

**Published:** 2014-07-10

**Authors:** Jason M. Wolfs, Matthew DaSilva, Sarah E. Meister, Xu Wang, Caroline Schild-Poulter, David R. Edgell

**Affiliations:** 1 Department of Biochemistry, Schulich School of Medicine and Dentistry, Western University, London, ON, N6A 5C1, Canada; 2 Robarts Research Institute, Schulich School of Medicine and Dentistry, Western University, London, ON, N6A 5B7, Canada

## Abstract

Targeting gene disruptions in complex genomes relies on imprecise repair by the non-homologous end-joining DNA pathway, creating mutagenic insertions or deletions (indels) at the break point. DNA end-processing enzymes are often co-expressed with genome-editing nucleases to enhance the frequency of indels, as the compatible cohesive ends generated by the nucleases can be precisely repaired, leading to a cycle of cleavage and non-mutagenic repair. Here, we present an alternative strategy to bias repair toward gene disruption by fusing two different nuclease active sites from I-TevI (a GIY-YIG enzyme) and I-OnuI E2 (an engineered meganuclease) into a single polypeptide chain. *In vitro*, the MegaTev enzyme generates two double-strand breaks to excise an intervening 30-bp fragment. In HEK 293 cells, we observe a high frequency of gene disruption without co-expression of DNA end-processing enzymes. Deep sequencing of disrupted target sites revealed minimal processing, consistent with the MegaTev sequestering the double-strand breaks from the DNA repair machinery. Off-target profiling revealed no detectable cleavage at sites where the I-TevI CNNNG cleavage motif is not appropriately spaced from the I-OnuI binding site. The MegaTev enzyme represents a small, programmable nuclease platform for extremely specific genome-engineering applications.

## INTRODUCTION

The rapid pace of development in the genome-editing field has led to a number of competing technologies, each with their benefits and limitations (1,2). The technologies can be broadly characterized based on the nuclease domain used to introduce a double-strand break (DSB) or nick at a target site. Two common reagents are zinc-finger nucleases (ZFNs) and transcription activator-like effector nucleases (TALENs) that utilize the dimeric and non-specific FokI nuclease domain (3–6). Two head-to-head ZFN or TALEN pairs must be designed to target a single site and positioned such that the FokI domains can dimerize to introduce a DSB (7–9), typically with 4-nt 5′ overhangs. The non-specific cleavage activity of the FokI nuclease domain facilitates targeting of a wide range of sequences, but can lead to off-target cleavage (10–12). The recently developed CRISPR/Cas9 system has received significant attention due to the ease of programming targeting (13,14). In this system, a ribonucleic acid (RNA) guide molecule (the crRNA) targets the Cas9 nuclease to a deoxyribonucleic acid (DNA) target through an RNA/DNA heteroduplex (15–18). A blunt-ended DSB results from two independent nicking reactions, one by a HNH nuclease domain and the other by a RuvC-like domain. An alternative nuclease architecture utilizes the naturally occurring meganucleases, or LAGLIDADG family homing endonucleases, which are typically encoded within self-splicing group I introns and inteins, and are characterized by an extensive protein–DNA interface (19,20). The nuclease active site is formed at the interface of two parallel α-helices, with cleavage generating a DSB with 4-nt 3′ overhangs. Recently, a number of recombinase (21,22) and sequence-specific nuclease domains have been developed as alternatives to the non-specific FokI nuclease domain (23–26). In particular, we and others showed that the monomeric and sequence tolerant GIY-YIG nuclease domain from the homing endonuclease I-TevI could be fused to zinc fingers, meganucleases and TAL effectors to create novel monomeric enzymes (27,28). The I-TevI-based reagents are active on substrates that contain a preferred CNNNG cleavage motif, generating 2-nt 3′ overhangs.

Regardless of the technology, one common application of genome-editing nucleases is the generation of gene disruptions whereby mutagenic repair at the targeted DSB introduces frameshift mutations into a coding region. The mutagenic DNA repair events occur in the absence of an exogenously provided DNA repair template and result mainly

*To whom correspondence should be addressed: Tel: +1 519-661-3133; Fax: +1 519-661-3175; Email: dedgell@uwo.ca from the non-homologous end-joining (NHEJ) pathway (29–31). However, many DSBs are repaired without mutation, as the compatible cohesive ends generated by the nucleases are re-ligated through the canonical NHEJ pathway, leading to a cycle of persistent cleavage and precise repair events that are non-productive for genome engineering. One strategy to bias repair events toward gene disruption is to co-express a DNA end-processing enzyme with the genome-editing nuclease (32,33). For instance, Trex2, a 3′–5′ exonuclease, dramatically increases gene disruption when co-expressed with ZFNs, TALENs and meganucleases by processing of DSBs before DNA repair. One potential limitation of this strategy is the requirement to transfect the Trex2 coding region with the ZFN, meganuclease or dimeric TALEN constructs, which may be problematic in size-constrained vectors. Overexpression of Trex2 could also enhance mutagenic repair at unwanted off-target sites, although no increases in cellular toxicity or off-target cleavages were observed with Trex2 over-expressing cell lines (32,33). Gene disruption could be enhanced by targeting two reagents to the same locus, positioning two DSBs to effectively excise the intervening sequence and introduce a deletion. Such multiplexing of genome-editing regents is constrained by the dimeric architecture of the ZFNs and TALENs (34) and, in the case of the CRISPR/Cas9 system, requires the use of nicking variants and dual-guide RNAs (35,36).

Here, we propose an alternative strategy for gene disruption by coupling two different nuclease active sites into a single polypeptide. The MegaTev architecture is the fusion of a meganuclease (Mega) with the nuclease domain derived from the GIY-YIG homing endonuclease I-TevI (Tev). The two active sites are positioned ~30 bp apart on DNA substrate, and generate two DSBs with non-compatible cohesive ends. The dual active MegaTev shows high gene disruption activity in HEK 293 cells without overexpression of DNA-end processing enzymes.

## MATERIALS AND METHODS

### Bacterial strains and plasmid construction

*Escherichia coli* DH5α (New England Biolabs) was used for plasmid amplification, ER2566 (New England Biolabs) for protein expression and BW25141 (λDE3) for bacterial two-plasmid selections (Supplementary Table S1) (37). Tev-Onu and Tev-Ltr fusions were cloned into pACYCDuet-1 using 5′ NcoI and 3′ XhoI sites as previously described (27). For the yeast DNA repair assay (3,38), the *Tev-Onu* and *Tev-Ltr* genes were amplified using Phusion DNA polymerase (New England Biolabs) with a 3′ primer that introduced a C-terminal SV40 nuclear localization sequence (NLS) (primers are listed in Supplementary Table S2). Polymerase chain reaction (PCR) products were cloned into the NcoI/SalI sites of pGPD. The backbone target site plasmid for the yeast assay was created by amplifying a 300-bp fragment of the pTox plasmid, digesting the fragment with BglII/SpeI, and cloning the digested fragment into pCP5.1 digested with BglII/SpeI to create pCPTox. All target sites were subsequently cloned into pCPTox using *in vivo* homology directed repair. For mammalian assays, human codon optimized Tev-Onu fusions (synthesized by IDT-DNA) were PCR amplified and cloned PstI and RsrII into pExodus. Tev-Onu fusions were cloned in-frame with a *mCherry* gene linked by a T2A peptide sequence from *Thosea asigna* virus to separate the translated proteins. The TO15 target site was subcloned into the pMSCVpuro retroviral vector (Clontech) using BglII and XhoI sites to integrate into genomic DNA in HEK 293 cells. To generate target sites for episomal plasmid assays, substrates were cloned into the SacI/XhoI sites of pcDNA3(+) vector. Constructs were confirmed by sequencing.

### *In vitro* randomized substrate selection

A list of randomized target site oligonucleotides is found in Supplementary Table S2. The target site plasmid library for the randomized cleavage motif plus 3 bp of the spacer (N8) were constructed in the pSP72 backbone as described (39). The library complexity was estimated to be ~6.4 x $10^4$ for the N8 library based on the number of independent transformants, and from analyses of next-generation sequencing data.

Cleavage assays were performed with 23 nM of Tev169-Onu E22Q and 10-nM N8 plasmid in NEBuffer 3 (50-mM Tris-HCl pH 7.9, 100-mM NaCl, 10-mM $MgCl_2$ and 1-mM dithiothreitol (DTT) ) at 37°C for 5 min. The Tev169-Onu fusions were purified as described (27) (Supplementary Figure S1). Samples were prepared for Ion Torrent sequencing at the London Regional Genomics Centre by PCR amplification of the target site region from the input plasmid library and from the plasmids isolated after three rounds of selection using PWO DNA polymerase (Roche) with barcoded primers. The sequencing data were parsed with custom Perl scripts that checked for anchor sequences either side of the randomized region, confirmed that the sequence between the anchors corresponding to the randomized region was 8 nt in length, and then extracted the randomized region for further analyses. For each round of selection, counts for each nucleotide *j* per position *i* were determined and then converted to proportions using the centered log-ratio transformation:

$$C_{i,j} = \log_2(p_{i,j}) - \text{mean } \log_2(p_j).$$

Nucleotide selection was then determined by taking the difference in proportions for each nucleotide per position between the final round of selection and the input library. A positive value indicates selection or enrichment for a particular nucleotide relative to the input library, and a negative value indicates selection against a particular nucleotide relative to the input. The enrichment values were plotted in heat map format using R and ggplot2 (40,41), and enrichment values were considered significant if they were >2 standard deviations from the mean enrichment value for each data set.

### Cleavage assays on radiolabeled substrate

*In vitro* cleavage assays were performed on internally radiolabeled substrates that were PCR amplified with [α-$^{32}$P] dCTP. PCR products were loaded onto an 8% (w/v) polyacrylamide gel (29:1 acrylamide/bisacrylamide), run at 40 mA for ~1.5 h, gel purified, eluted overnight at 42°C in

5 ml of TE pH 8.0 and concentrated into 50-μl volume. Cleavage reactions were performed in 20-μl reaction volumes with NEBuffer 3 (50-mM Tris-HCl pH 7.9, 100-mM NaCl, 10-mM $MgCl_2$ and 1-mM DTT), 0.1 pmol of substrate and 2 pmol of Tev169-Onu fusion protein. Cleavage reactions were incubated at 37°C for 1, 5, 10 and 25 min before stopping the reaction with 6 μl of 100-mM ethylenediaminetetraacetic acid (EDTA) and 5 μl of loading dye containing 0.5% sodium dodecyl sulphate (SDS). Mutant Tev169-Onu fusions were incubated for 1 h at 37°C before stopping the reaction with 6 μl of 100-mM EDTA and 5 μl of loading dye containing 0.5% SDS. The entire reaction was loaded on a 15% (w/v) polyacrylamide gel (29:1 acrylamide/bisacrylamide) and electrophoresed at 40 mA for ~1.5 h. The gel was removed from the apparatus and soaked in 10% glycerol plus 8% acetic acid before drying on Whatman paper and visualized using a phosphorimager (GE Healthcare).

### Modified two-plasmid target site screen

The 64-triplet variants for the CNNNG cleavage motif were screened using a modified two-plasmid selection (42). Transformants were gridded onto selective plates (LB plus 25-μg/ml chloramphenicol and 10-mM L-(+)-arabinose) and non-selective plates (Luria Broth plus 25-μg/ml chloramphenicol, 50-μl/ml kanamycin and 0.2% glucose) and incubated overnight at 37°C. Plasmids isolated from survivors and non-survivors were sequenced to identify the NNN variant of the CNNNG motif.

### Yeast β-galactosidase repair assay

This assay was performed as described (27). Briefly, YPH499(a) containing target site constructs were mated in triplicate with YPH500(α) harboring the MegaTev constructs. After an overnight selection for diploids, cells were assayed for β-galactosidase activity using orthonitrophenol (ONPG). Activity was normalized to either a validated homodimeric zinc-finger nuclease (Zif268) or the wild-type TP15 substrate depending on the assay.

### DdeI-resistance assays with plasmid substrates

HEK 293T cells were cultured in Dulbecco's modified Eagle's medium supplemented with 10% fetal bovine serum. Approximately $2.5 \times 10^6$ million cells were seeded 24 h before transfection on 6-cm plates. Cells were co-transfected with 3 μg of pExodus Tev169-Onu E22Q and 3 μg of pcDNA3(+) TO15 using calcium phosphate and incubated at 37°C with 5% $CO_2$ for 16 h before changing media. After 48 h, plasmid was isolated from HEK 293T cells using the BioBasic miniprep kit. Target sites were PCR amplified, separated on a 1% agarose gel and gel purified. After gel purification, 250 ng of PCR product was incubated with 2 U of DdeI (New England Biolabs) in NEBuffer2 for 1 h at 37°C. Digests were electrophoresed on a 1.5% agarose gel, stained with ethidium bromide and analyzed on an AlphaImager™ 3400 (Alpha Innotech).

### Surveyor assays with integrated targets

Target site integration into HEK 293 cells was performed using the Phoenix Ampho retroviral packaging cell line. To accomplish this, 8 μg of pMSCV TO15 was transfected into Phoenix cells using calcium phosphate and incubated at 37°C with 5% $CO_2$ for 48 h. Media was removed and filtered through a 0.45-μm filter into a falcon tube containing 6 μl of 4-mg/ml polybrene (hexadimethrine bromide), and 6 ml of virus solution was used to infect HEK 293 cells to create the integrated cell line (HEK 293-TO15). Approximately 24 h before transfections, $\sim 2.5 \times 10^6$ HEK 293-TO15 cells were seeded on 6-cm plates and subsequently transfected with 6 μg of pExodus Tev169-Onu or pExodus I-SceI. After 48 h, the HEK 293-TO15 cells were harvested and total genomic DNA isolated. Two rounds of nested PCR were performed, and gel purified PCR products were boiled at 95°C for 10 min, then cooled slowly to 50°C before flash freezing at −20°C for 2 min. To assay for indels, 200 ng of PCR product was incubated with 2 U of T7 endonuclease I (New England Biolabs) in NEBuffer2 for 1 h at 37°C, separated on a 1.5% agarose gel and analyzed using an AlphaImager™ 3400 (Alpha Innotech).

### Western blots

Whole cell extracts were prepared 16 h post-transfection in lysis buffer (50-mM HEPES pH 7.4, 150-mM NaCl, 1-mM EDTA, 10% glycerol and 0.5% NP40) supplemented with protease and phosphatase inhibitors. After 30-min incubation on ice, lysates were centrifuged for 10 min, and the supernatant transferred to a new tube. One hundred micrograms of supernatant was loaded on a 12% SDS-polyacrylamide gel electrophoresis followed by a 1-h transfer to a polyvinylidene difluoride membrane and overnight hybridization at 4°C with an HA (HA-7, Sigma) antibody. Blots were developed using the Western Lightning® Enhanced Chemiluminescence Reagent (Perkin Elmer, Waltham, MA, USA). Quantifications were done using ImageJ software and Image Lab (BioRad, Hercules, CA, USA).

### Caspase assays

HEK293 cells were harvested 48 h post-transfection or post-treatment with 100-μM etoposide. Cell extracts were prepared in lysis buffer (10-mM HEPES pH 7.4, 1-mM KCl, 1.5-mM $MgCl_2$, 10% glycerol, 5-μg/ml aprotinin, 1-μM DTT, 2-μg/ml leupeptin, 0.2-mg/ml phenylmethylsulfonyl fluoride and 10% NP40). After 15-min incubation on ice, cell lysates were centrifuged for 10 min and the supernatant transferred to a new tube. Caspase-3 activity was measured using 2 μg of extract in caspase assay buffer (25-mM HEPES pH 7.5, 10-mM DTT, 10% sucrose, 0.1% CHAPS containing 10-μM caspase-3 substrate *N*-acetyl-Asp-Glu-Val-Asp-(7-amino-4trifluoromethyl-couarin) (DEVD-AFC). Fluorescence was measured in a 96-well plate using a SpectraMax M5 fluorimeter (excitation 400 nm, emission 505 nm) with readings every 15 min for 2 h.

### Illumina sequencing

Target sites were amplified with barcoded primers, pooled and sequenced on an Illumina Mi-Seq platform at the London Regional Genomics Centre at Western University. The biological replicates were sequenced on two independent runs. Reads were processed for valid barcodes, and for the presence of primer sequences that flanked the MegaTev binding site using custom Perl scripts. Primer sequences were removed from parsed reads, the length and abundance determined and the data analyzed and plotted in R (41) using the ggplot2 package (40).

## RESULTS

### MegaTevs: chimeric fusions of GIY-YIG and meganuclease components

To determine if the I-TevI nuclease domain could function in the context of different meganucleases, we fused residues 1–169 of I-TevI (Tev169) to the native N-terminus of the catalytically inactive I-LtrI variant, I-LtrI E29Q, to create Tev169-Ltr E29Q (Tev-xLtr) (Figure 1A). Along with the previously constructed Tev169-Onu E22Q (Tev169-xOnu) (27), we assayed the activity of both MegaTevs using a yeast recombination assay where a target site is positioned between a partially duplicated *lacZ* gene. Cleavage of the target site induces the single-strand annealing pathway to reconstitute a functional *lacZ* gene resulting in β-galactosidase activity. We tested activity on hybrid target sites consisting of the native I-TevI CNNNG cleavage motif (5′-CAACG-3′) and DNA spacer derived from the phage T4 thymidylate synthase (*td*) gene fused to either the I-OnuI E2 or the I-LtrI binding site (TO or TL, respectively) (Figure 1A). The substrates differed in the length of the DNA spacer (from 11 to 21 bp) separating the I-TevI CNNNG cleavage motif from the I-OnuI E2 or the I-LtrI binding site. As shown in Figure 1B, Tev169-xOnu and Tev169-xLtr activity was highest with a DNA spacer length of 15 bp, agreeing with results from profiling DNA spacer length requirements of the Tev-xOnu construct in a bacterial two-plasmid survival assay (27). Furthermore, mutating the critical cleavage CNNNG motif to ANNNA abolished activity for both Tev169-xOnu and Tev169-xLtr on the 15-bp spacer substrate [TO15CS(−) and TL15CS(−)], demonstrating that the I-TevI nuclease domain maintains cleavage specificity in the context of a meganuclease fusion. To demonstrate that the MegaTevs are directed to their target sites by the meganuclease and not the I-TevI nuclease domain and linker, we tested the Tev-xOnu against the TL15 target site, and tested the Tev-xLtr against the TO15 target site. No activity was observed for either fusion on the reciprocal substrates (Figure 1B), showing that the I-TevI nuclease domain does not direct targeting of the MegaTevs.

### Dual active site MegaTevs for highly efficient targeted deletions

A unique aspect of the MegaTevs is the fusion of two homing endonuclease active sites into a single polypeptide chain. Each active site is positioned such that the top-strand nicking sites are separated by ∼30 bp on the TO15 DNA substrate. When both active sites are functional, this arrangement presents the opportunity to introduce two DSBs with different cohesive ends at a single site in a highly efficient and concerted process. As a proof of concept, we constructed and purified a Tev169-Onu dual nuclease where both the I-TevI and I-OnuI E2 active sites are wild type (Tev169-Onu; Figure 1C and Supplementary Figure S1). Activity was tested *in vitro* utilizing an internally radiolabeled PCR product of 242 bp containing the TO15 target site. Cleavage by the dual nuclease would be evident by the release of 29-bp product corresponding to the internal sequence between the I-TevI and I-OnuI E2 cleavage sites (Figure 1C). As shown in Figure 1D, the dual active MegaTev efficiently produced three products after 5 min of digestion, with the accumulation of the internal product (IP) after 25 min. Interestingly, cleavage by the I-TevI nuclease domain precedes cleavage by I-OnuI E2, as I-TevI-specific products are detected at 1 min (TP1 and TP2), whereas I-OnuI E2 products are detected after 5 min (OP1 and OP2). Cleavage assays where I-TevI is active and I-OnuI E2 is inactive (Tev169-xOnu) produced two products consistent with only I-TevI cleavage activity. Similarly, a catalytically inactive R27A I-TevI in the context of an active I-OnuI E2 fusion (xTev169-Onu) produced two products consistent with I-OnuI E2 cleavage. No cleavage was observed for the dual dead nuclease (inactive I-TevI R27A and I-OnuI E22Q, xTev-xOnu) after 1 h of incubation. We also constructed and purified an analogous Tev169-Ltr dual nuclease (Supplementary Figure S1) and tested for activity on an internally labeled PCR product containing the TL15 site (Supplementary Figure S2). I-TevI-specific cleavage products were observed before I-LtrI products, with the internal cleavage product visible after 5 min of incubation.

### Expression and activity of MegaTevs in HEK 293 cells

To test expression and integrity of the MegaTevs in human cell lines, we constructed codon-optimized versions. The expression constructs included the MegaTev open-reading frame (ORF), followed by an in-frame T2A peptide and mCherry ORF, allowing us to monitor MegaTev expression by mCherry levels (Figure 2A). Robust expression was observed for all MegaTev constructs. We also tagged the C-terminus of the MegaTev fusion and the I-OnuI E22Q enzyme with an hemagglutinin (HA) epitope tag to monitor protein integrity by western blot and found that ∼72% of the MegaTev enzyme was full length 16 h post-transfection (Figure 2B). A smaller sized band of ∼45 kDa was present in western blots. The size of this product is consistent with proteolytic cleavage within the I-TevI linker region of the MegaTev construct. In contrast, MegaTevs with an HA tag at the N-terminus could not be detected by western blot. Proteolytic cleavage of the MegaTev enzyme to release N-terminal fragments consisting of the I-TevI nuclease domain could potentially generate nuclease domains with non-specific activity, although previous studies have shown that the GIY-YIG catalytic domains have very low affinity for DNA. To rule out potential toxic side effects associated with non-specific nuclease activity of the I-TevI catalytic domain, we overexpressed the Tev1-169 domain in HEK 293 cells. After 48 h post-transfection, we isolated cell

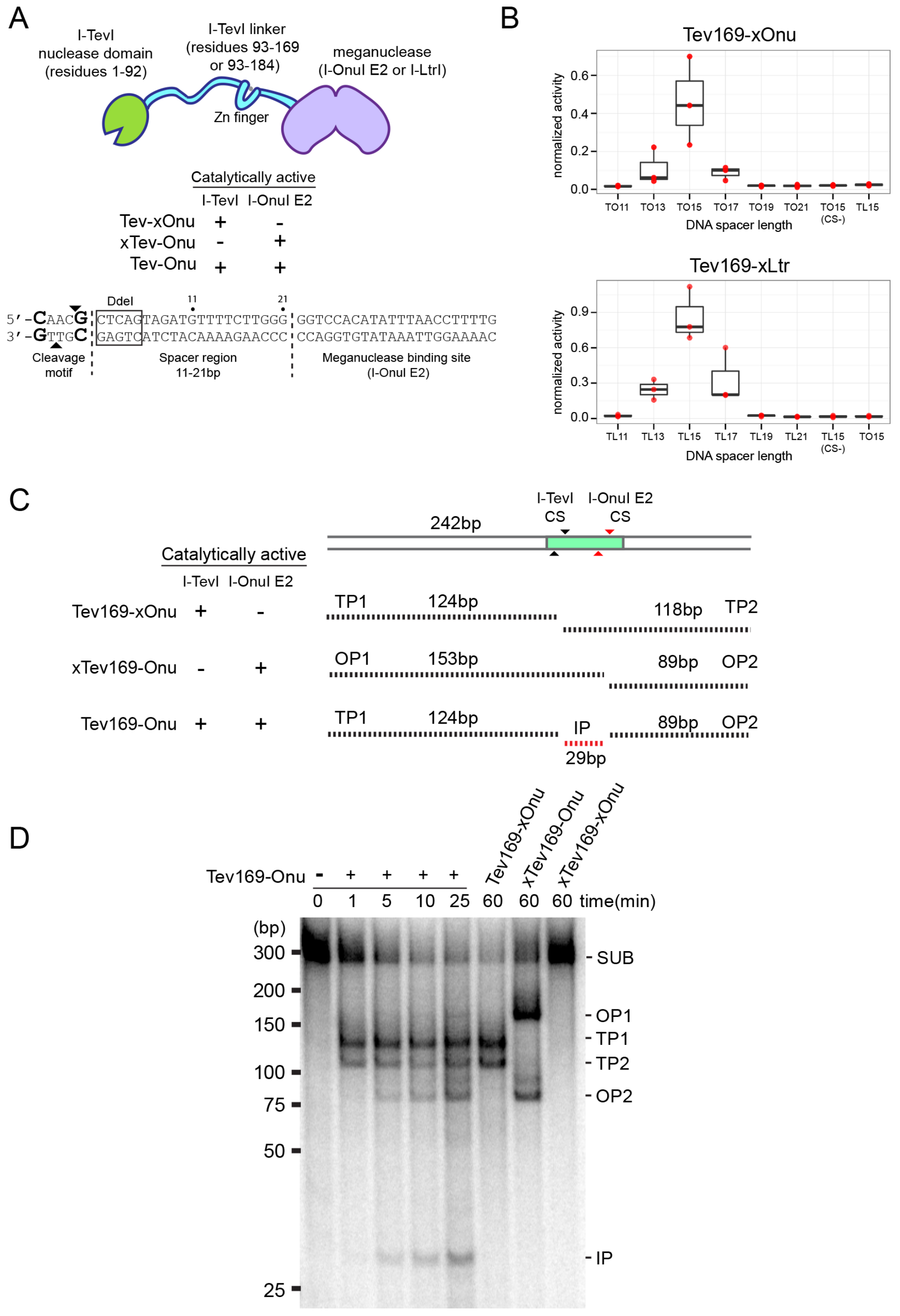

**Figure 1.** MegaTev activity *in vitro* and in yeast. (**A**) (top) Schematic for the modular MegaTev fusions, consisting of the I-TevI nuclease domain and various linker lengths fused to a meganuclease. Catalytically inactive R27A I-TevI and E22Q I-OnuI E2 are denoted by xTev and xOnu, respectively. (Bottom) The modular composition of the target site consisting of the 5′-CNNNG-3′ (CAACG) cleavage motif and variable length spacer region (11–21 bp) from the native I-TevI thymidylate synthase (*td*) gene located upstream of the meganuclease binding site. The arrows highlight the top and bottom I-TevI cleavage sites. Highlighted in the box is the DdeI site adjacent to the I-TevI cleavage site used to identify mutagenic events in HEK 293T cells. (**B**) Boxplot of the activity for Tev169-xOnu (top) and Tev169-xLtr (bottom) on various DNA spacer lengths in the yeast recombination assay. TO15CS(−), AAACA cleavage motif in the TO15 target site; TL15CS(−), AAACA cleavage motif in the TL15 target site; xLtr, a catalytically inactive E29Q I-LtrI. Activity was normalized to the homodimeric FokI-Zif268 fusion and data are plotted with SD for $n = 3$. (**C**) Schematic of substrate and cleavage products containing the TO15 target site indicated by a shaded rectangle. The black and grey arrows indicate the top and bottom cleavage sites (CS) for I-TevI and I-OnuI E2, respectively. Black dashed lines indicate I-TevI cleavage products (TP1 and TP2), I-OnuI E2 cleavage products (OP1 and OP2), and the internal product (IP) from both I-TevI and I-OnuI cleavages. Sizes of the substrate and products are indicated in base pairs (bp). (**D**) Polyacrylamide gel of internally labeled TO15 PCR substrate (SUB) incubated with (+) or without (−) purified dual active Tev169-Onu and single active site variants. The MegaTevs were incubated with substrate for the indicated times, and cleavage products are indicated on the right side of the gel based on their predicted sizes from panel (C). The sizing standard (in bp) was cropped from the gel image.

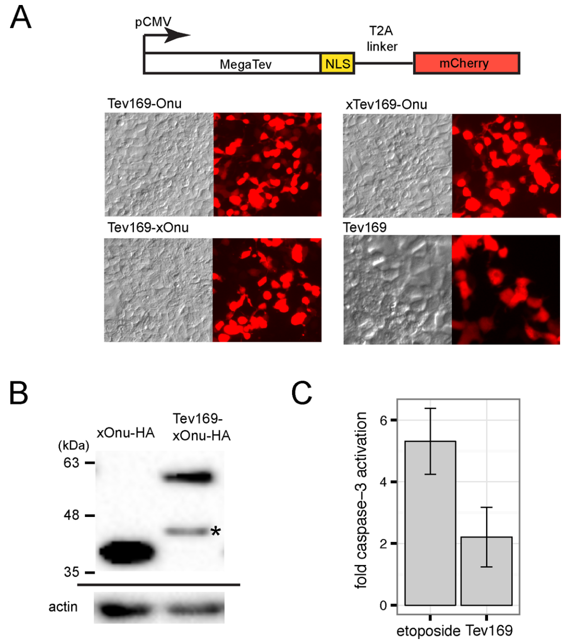

**Figure 2.** MegaTev expression and integrity in HEK 293 cells. (**A**) Schematic of the expression construct, with the MegaTev ORF linked to mCherry through a T2A peptide. The cytomegalovirus (CMV) promoter was used to express the constructs. NLS, nuclear localization sequence. Representative contrast and fluorescence images for cells transfected with the indicated MegaTev fusions are shown below. (**B**) Western blot of HEK 293 whole cell extracts isolated 16 h post-transfection with C-terminally HA-tagged versions of I-OnuI E22Q or Tev169-xOnu. Size standards are indicated on the left of the gel image. The blot was stripped and reprobed with an actin antibody (shown below). The asterisk indicates a smaller fragment that is presumed to be a proteolytic fragment of Tev169-xOnu. (**C**) Caspase-3-fold activation in HEK 293 cell extracts treated with etoposide or from extracts of HEK 293 cells overexpressing the Tev169 nuclease domain fragment. Data are plotted as the average of three biological replicates normalized to caspase-3 activity in HEK 293 cells transfected with an eGFP expression plasmid. Error bars represent the standard deviation of the mean.

extracts and monitored caspase-3 activity as an indicator of DNA damage response. As a positive control, HEK 293 cells were treated with etoposide to induce DNA damage. No significant difference was observed in caspase-3 activity for HEK 293 cells transfected with the Tev169 or the eGFP expression plasmids, whereas significant caspase-3 activity was found in extracts of HEK 293 cells that were incubated with etoposide (Figure 2C). Collectively, these data demonstrate that MegaTevs are robustly expressed with low levels of toxicity in HEK 293 cells.

### MegaTevs induce mutagenic repair in HEK 293 cells

To extend the utility of the MegaTev constructs to an *in vivo* context, we first co-transfected the single nuclease variant, Tev169-xOnu, and the TO15 target site cloned on a separate plasmid into HEK 293T cells. To monitor Tev-xOnu activity, we took advantage of a DdeI site that lies immediately downstream of the I-TevI CNNNG motif (Figure 1A). Plasmid substrates cleaved *in vivo* by the MegaTev would be subject to mutagenic non-homologous end-joining repair, destroying the DdeI site. Subsequent resistance of target sites to DdeI digestion after PCR amplification from genomic DNA reflects Tev-xOnu cleavage and mutagenic repair. Significantly, we observed 9% DdeI cleavage resistance for target sites amplified from HEK 293T cells co-transfected with Tev-xOnu and the TO15 target site (Figure 3A). In contrast, no DdeI-resistant products were observed for cells transfected with the TO15 target site plasmid only. To confirm that DdeI resistance assay was detecting Tev169-xOnu events derived from cleavage and mutagenic repair of plasmid substrates, we transformed *E. coli* cells with total DNA isolated from HEK 293 to enrich for circular plasmids. Plasmid DNA was prepared in bulk, the TO15 target PCR amplified and subsequently digested with DdeI revealing ∼9% cleavage resistance (Supplementary Figure S3). Cleavage-resistant products were cloned and sequenced, revealing deletions that spanned the I-TevI cleavage site (Supplementary Figure S3), demonstrating that MegaTevs function to induce mutagenic DNA repair in human cells.

Next, we stably integrated the TO15 target site into the genome of HEK 293 cells and assayed for activity after independent transfections with the Tev169-xOnu and xTev169-Onu constructs. After 48 h of incubation, the TO15 target site was PCR amplified and activity was assessed in two ways. First, the PCR products were digested with T7 endonuclease I (T7EI). T7EI was used rather than DdeI digestion to allow us to detect I-OnuI E2-specific events with the xTev169-Onu construct. As shown in Figure 3B, undetectable levels of cleavage were observed with both of the single active site Tev169-Onu fusions (xTev169-Onu and Tev169-xOnu), in spite of similar expression levels (Figure 2A). Second, the amplified TO15 target site from two independent transformations of the nucleases was deep sequenced using the Illumina Mi-Seq platform. Activity was determined as the number of reads that possessed indels relative to the wild-type TO15 sequence (Table 1). We observed an ∼4–6% indel rate with the Tev169-xOnu variant over two experimental replicates, consistent with the indel rate estimated from DdeI cleavage resistance assays. Deep sequencing revealed a spectrum of deletion phenotypes, most of which were centered on the I-TevI cleavage site (Figure 3C). In contrast, a <1% indel rate with the xTev169-Onu single active site variant was indistinguishable from the indel rate at the TO15 site sequenced from mock-transfected cells. Extremely low levels of cleavage by the xTev169-Onu variant, where I-OnuI E2 is active, are consistent with previous studies that required sorting for cells with high I-OnuI E2 expression levels and multiple rounds of PCR enrichment to visualize I-OnuI E2 cleavage (43).

### Dual active MegaTevs efficiently induce deletions on integrated targets

In contrast to the single active site variants, higher activity was detected for the dual active MegaTev enzyme on the integrated TO15 target site (Figure 3B). Between 20 and 40% activity was inferred from T7E1 assays on amplified PCR target sites from transfections of the Tev169-Onu dual nuclease, and from cells transfected with another MegaTev construct containing the I-TevI 1-184 fragment fused to I-OnuI E2 (Tev184-Onu). To confirm that MegaTev activity resulted in deletion of the sequence between the I-TevI and I-OnuI E2 cleavage sites, we sequenced individual cloned

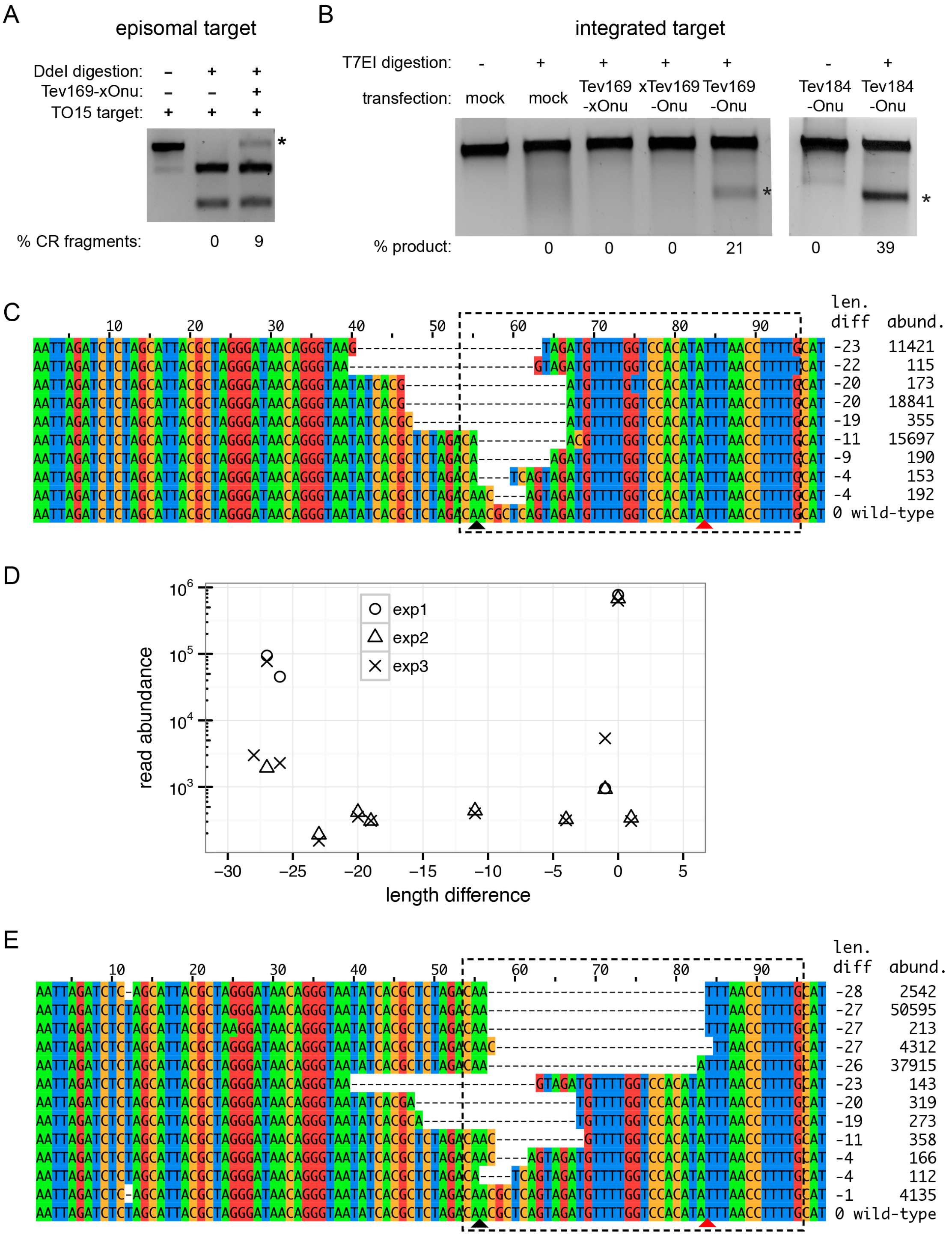

**Figure 3.** MegaTev activity in HEK 293 cells. (**A**) DdeI digestion of PCR products amplified from episomal substrates to identify mutagenic events in HEK 293T cells. Shown is a representative agarose gel for DdeI-digested-PCR-amplified target sites from plasmids isolated 48 h post-transfection. The DdeI cleavage-resistant product is indicated by the asterisk, with percent product indicated below each lane. (**B**) T7 endonuclease I (T7EI) assays on integrated TO15 target sites. Shown are representative digests of TO15 target site PCR products from HEK 293 cells transfected with the indicated MegaTevs. The extent of T7EI digestion is indicated below each lane. An asterisk indicates the T7EI cleavage products. (**C**) Representative reads from Illumina sequencing of TO15 target sites amplified from HEK 293 cells transfected with the Tev169-xOnu fusion. The length difference of each read relative to the wild-type TO15 sequence (98 nts) is indicated on the right, as is the abundance of each read in the sequencing data. The MegaTev binding sites are indicated by a dashed box, and the bottom-strand nicking sites of I-TevI and I-OnuI are indicated by black and red triangles, respectively. (**D**) Plot of length difference versus read abundance for Illumina sequencing data of the TO15 target site amplified from three independent transfections of the dual active Tev169-Onu MegaTev into HEK 293 cells. Length difference is relative to the wild-type TO15 sequence, represented as 0 in this plot. Reads that occurred more than 100 times are plotted. (**E**) Representative Illumina reads from the dual active MegaTev transfections. The length difference and read abundance are indicated to the right, and the dashed box indicates the MegaTev binding site. The bottom-strand nicking sites of I-TevI and I-OnuI are indicated by black and red triangles, respectively.

**Table 1.** Indel rates of Tev-Onu nuclease variants determined from Illumina sequencing

| Target site | Nuclease | Replicate | Total reads | Reads with indels (%) |
|---|---|---|---|---|
| TO15 integrated | Tev169-xOnu | 1 | 968810 | 64054 (6.6) |
| | | 2 | 1265885 | 47457 (3.7) |
| | xTev169-Onu | 1 | 913179 | 5947 (0.6) |
| | | 2 | 891255 | 5914 (0.7) |
| | Tev169-Onu | 1 | 911588 | 140843 (15.5) |
| | | 2 | 719679 | 90329 (12.6) |
| | Mock | | 611167 | 4322 (0.7) |
| MAO-B | Tev169-Onu | 1 | 344322 | 4868 (1.4) |
| | | 2 | 236485 | 4203 (1.8) |
| | Mock | | 327880 | 4092 (1.2) |
| Off-target #3 | Tev169-Onu | 1 | 718999 | 1749 (0.2) |
| | | 2 | 782299 | 110 (0.01) |
| | | 3 | 518139 | 1085 (0.2) |
| Off-target #4 | Tev169-Onu | 1 | 1467447 | 5025 (0.3) |
| | | 2 | 959483 | 2770 (0.3) |
| | | 3 | 1012599 | 3124 (0.3) |
| | Mock | | 949836 | 2718 (0.3) |
| Off-target #15 | Tev169-Onu | 1 | 1192183 | 1661 (0.1) |
| | | 2 | 900634 | 2561 (0.3) |
| | Mock | | 919388 | 1316 (0.1) |

PCR products and found three sequence types (M1, M2 and M3) that each occurred multiple times (Supplementary Figure S3). In each case, the intervening sequence between the I-TevI and I-OnuI E2 cleavage sites was deleted. To expand on these results, the PCR products from two independent transfections were analyzed by deep sequencing, revealing an indel rate of ∼12–15% (Table 1). Precise deletion of the intervening sequence between the I-TevI and I-OnuI E2 cleavage sites was confirmed by plotting the absolute length differences of the reads relative to the wild-type sequence (Figure 3D) and by examining individual reads from the deep sequencing data (Figure 3E). Smaller deletion lengths centered on the I-TevI cleavage were also observed (Figure 3E), but at a lower frequency (Figure 3D). The difference in estimated MegaTev activity between the deep sequencing data and T7E1 assays can be attributed to the fact that only length differences were counted as indels from the sequencing data, whereas the T7E1 assay will detect nucleotide mismatches in addition to indels.

Collectively, our data show that the fusion of two homing endonuclease active sites into a single polypeptide chain creates a dual nuclease that can introduce two DSBs at a single target site. In both an *in vitro* and *in vivo* context, the two DSBs excise a short internal fragment with high frequency. It is important to note that we did not co-express DNA end processing enzymes, such as the 3′–5′ exonuclease Trex2, to enhance mutagenic repair at the TO15 cleavage site.

## Requirements of the I-TevI CNNNG cleavage motif *in vivo*

Efficient cleavage by the I-TevI nuclease domain requires that the 5′-CNNNG-3‘ cleavage motif be spaced 15 bp from the meganuclease binding site (Figure 1B). Based on studies on the native enzyme, the C and G of the motif are critical for cleavage, while the central three bases (the NNN triplet) exhibit a substantial degree of tolerance to substitution (44,45). However, the tolerance of the central three bases to substitution in the context of the MegaTev fusion has not been assessed, and we used a variation of a two-plasmid bacterial selection to rapidly determine survival of all 64 variants of the central triplet cloned into the toxic plasmid. In this assay (42), the 64 toxic plasmids were transformed into cells harboring the Tev169-xOnu fusion, plated on non-selective plates, and then replica-gridded onto selective plates to induce expression of the *ccdB* gene on the toxic plasmid. Cells survive this challenge if Tev169-xOnu can cleave and promote elimination of the toxic plasmid. The Tev169-xOnu fusion was used for this experiment to ensure that survival was due to I-TevI activity and not I-OnuI activity. Three different morphologies were observed on the selective plates; no growth (dead), colonies that grew to the same diameter on both the selective and non-selective plates (strong survivors) and colonies that grew on selective plates but were smaller in diameter than on non-selective plates and often formed a cauliflower morphology (weak survivors) (Supplementary Figure S4). Weak survivors were determined to be target sites that promoted <1% survival when assayed individually. Survival was plotted in a heat map format (Figure 4A) revealing that C/G-rich triplets generally inhibited Tev169-xOnu survival.

We also assayed all 64-triplet variants in the quantitative yeast-based *lacZ* repair assay and plotted the activity for each triplet normalized to the activity of the wild-type AAC triplet on a $\log_2$ scale (Figure 4B). In general, more substitutions within the triplet resulted in lower activity, however some triplets with two or three substitutions were as active as the wild-type AAC triplet (Figure 4C). In particular, triplets with A or T at the first position display activity on par with the wild-type sequence (for instance, AAT, ATA, TAT and TTT). As with the bacterial two-plasmid selection, triplets with a C and/or G in the first two positions supported lower β-galactosidase activity, and triplets with a G in the third position were less active than other nucleotides at this position (Figure 4D).

## MegaTev selects for the appropriately spaced cleavage motif from a random substrate

To determine the optimal sequence and spacing of the cleavage motif, we generated a plasmid library where the CAACG cleavage motif and three downstream base pairs were completely randomized (Figure 5A, the N8 library). A round of *in vitro* selection with the N8 plasmid library consisted of *in vitro* digestion with purified Tev169-xOnu, isolation of linearized plasmid, re-ligation and transformation into *E. coli* for amplification. After three rounds of selection, the input library and the final round of selection were sequenced using the Ion Torrent platform. After data processing, we first determined the proportion of all 16 possible dinucleotide combinations (ANNNA, ANNNC, ANNNG, etc.) regardless of position within the N8-randomized region to ascertain if the MegaTev displayed a preference for the CNNNG motif. As shown in Figure 5A, the CNNNG

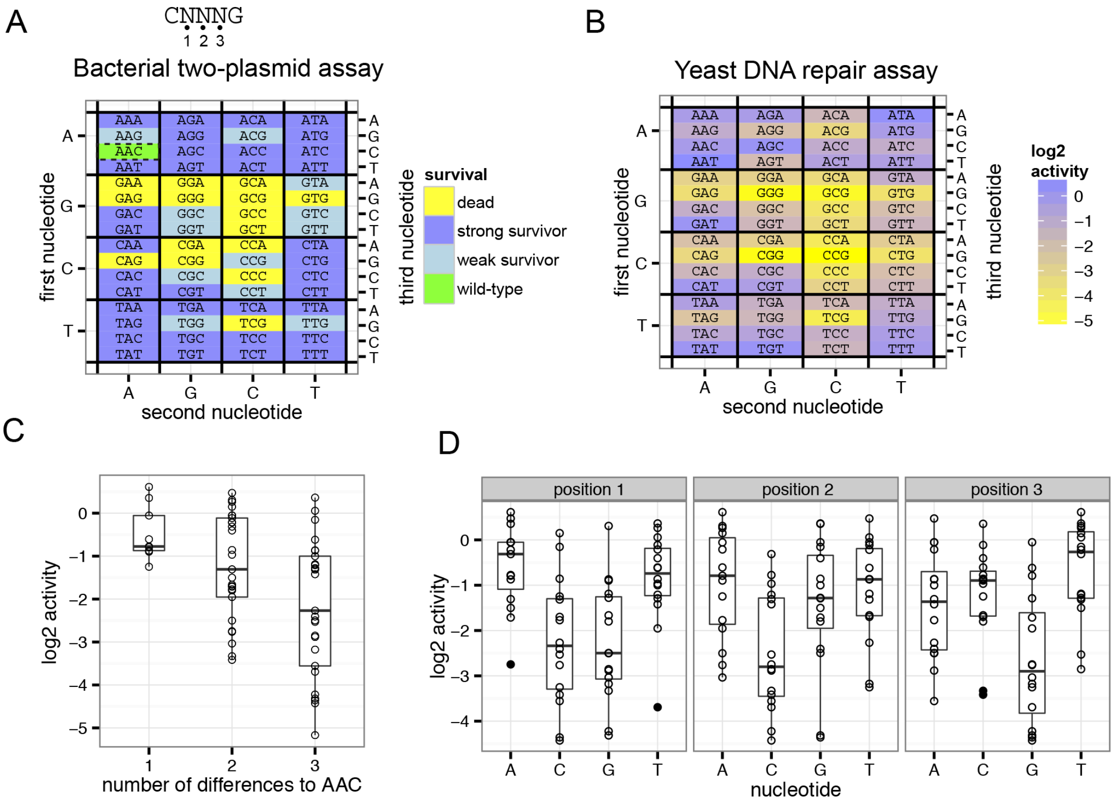

**Figure 4.** MegaTev nucleotide preference within the CNNNG cleavage motif. (**A**) The three nucleotides of the NNN triplet are labeled as 1, 2 and 3 from 5′ to 3′ within the CNNNG motif. Heat map of Tev169-xOnu activity on all 64 variants of the cleavage motif in the bacterial two-plasmid selection. The first nucleotide of the NNN triplet is plotted on the left axis, the second nucleotide of the NNN triplet on the bottom axis and the third nucleotide on the right axis. (**B**) Heat map of the activity of Tev169-xOnu on all 64-nucleotide variants in the yeast β-galactosidase repair assay. The values are normalized to the wild-type sequence (AAC) and plotted on a $\log_2$ scale. The heat map is labeled as in panel (A). (**C**) Boxplots depicting the $\log_2$ activity for NNN triplets that have 1, 2 or 3 nucleotide differences relative to the wild-type AAC sequence. For each boxplot, the upper and lower bounds of the boxes indicate the 25th and 75th percentile of the data, the solid horizontal bar indicates the median of the data and the ends of the whiskers represent 1.5 times the interquartile range. Individual data points are shown as open circles, and data points that lie outside of the interquartile range (outliers) are shown as black points. (**D**) Boxplots of the influence of nucleotide identity at each of the three positions within the NNN motif on activity relative to the wild-type AAC sequence. The boxplots are labeled as in panel (C).

motif was greatly enriched by round 1 relative to the other dinucleotide combinations, and predominated by round 3, indicating that CNNNG is the preferred motif. Although minor enrichment relative to the input library was observed for other dinucleotide combinations (ANNNA and ANNNT), these combinations do not support activity in cell-based assays, and are not considered relevant.

We next analyzed the phasing of the CNNNG motif within the N8-randomized region for reads containing this motif. This analysis was undertaken as native I-TevI can cleave the wild-type CAACG motif that has been moved closer to the primary binding site (albeit with lower efficiency) (46). The statistical occurrence of the CNNNG motif is 1 in 15 bp, and would be expected to occur at the four possible positions within the input N8 library (C1:G5, C2:G6, C3:G7 and C4:G8). Indeed, we observed the CNNNG motif with approximately equal frequency at the four possible positions within the input N8 library (Figure 5B, red bars). However, when the sequencing reads for selection rounds 1 and 3 were analyzed, we found an overwhelming preference for C and G at positions 1 and 5, respectively (Figure 5B, blue and gray bars). This analysis confirms that the MegaTev is cleaving at correctly positioned CNNNG motifs within the N8 library, with the G of the motif positioned 15 bp from the I-OnuI E2 binding site .

Using sequencing reads that possessed the CNNNG motif at positions 1 and 5 of the randomized region, we determined the abundance of each NNN triplet within the motif and plotted the $\log_2$ abundance in a heat map format (Figure 5C). As anticipated, A/T-rich triplets were preferred over G/C-rich triplets. This analysis also facilitated a comparison to the activity of each NNN triplet in the yeast DNA repair assay (Figure 4B). Plotting the normalized abundance of each NNN triplet from the sequencing data versus the activity in the yeast-based assay showed that A/T-rich NNN triplets supported higher activity relative to G/C-rich sequences in both data sets (Figure 5D).

## Nucleotide preference within the CNNNG motif and at flanking positions

We next analyzed nucleotide preferences at each position in the N8 library after the third round of selection. Nucleotide preferences were determined by calculating the proportional abundance of each nucleotide at each position for

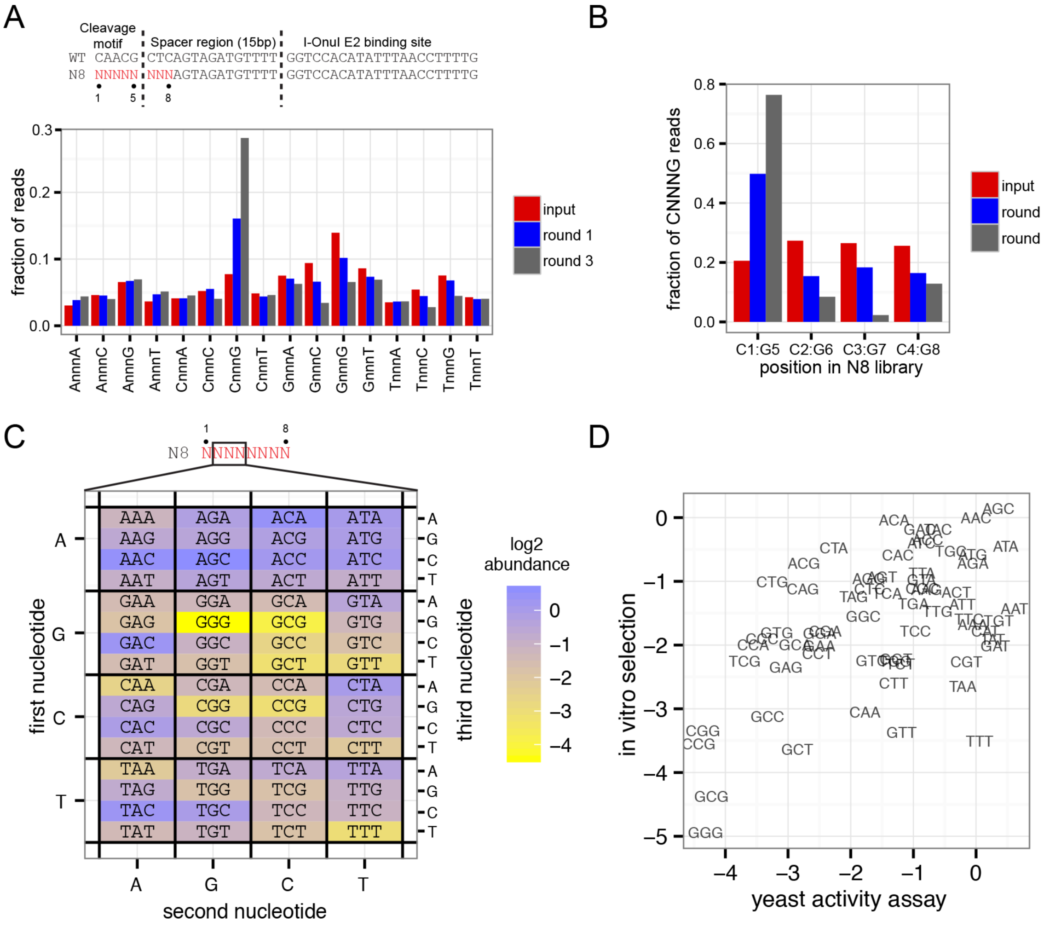

**Figure 5.** MegaTev selects for an appropriately spaced cleavage motif. (**A**) At top is a schematic of the TO15 substrate and N8-randomized library with the eight randomized positions colored in red. The randomized positions are labeled 1–8, with position 1 corresponding to the C of the CNNNG cleavage motif. Shown below is a bar graph showing the fraction of reads with each of the 16 possible dinucleotide combinations. (**B**) Bar graph showing the fraction of reads with the CNNNG cleavage motif at positions C1:G5, C2:G6, C3:G7 and C4:G8 for the input library and selection rounds 1 and 3. (**C**) Heat map plotted on a $\log_2$ scale showing abundance of all 64 CNNNG motifs after three rounds of selection with the Tev169-xOnu fusion. The first, second and third nucleotides correspond to the second, third and fourth nucleotides in the N8 library, respectively. (**D**) Correlation between the *in vitro* abundance from the selection experiments and *in vivo* activity in the yeast-based assay for each NNN triplet.

both the input library and round 3 selection, and then plotting the difference (enrichment) between round 3 and input as a heat map (Figure 6A). One advantage of this analysis is that it corrects for nucleotide bias in the input library. As shown in Figure 6A, apart from the expected C and G preference at positions 1 and 5, the strongest preference was observed at position 7, where T or A was selected for while a C or G was selected against. Interestingly, in four of the positions (3,6–8), the wild-type nucleotide was not preferred, implying that the native *td* target site of I-TevI is not the optimal substrate.

To provide an *in vivo* context for the nucleotide preferences observed in the flanking DNA sequence, we independently made point substitutions in the TO15 substrate at positions 6 and 7 and tested their activity in the yeast β-galactosidase assay (Figure 6B). Substitutions to A or T at position 6 did not drastically reduce activity as compared to the TO15 substrate, while the C6G substitution showed a modest increase in activity. At position 7, the T7A substitution reduced activity by half, while the T7G substitution reduced activity to background levels, supporting the enrichment preferences seen in the *in vitro* data.

Collectively, these data show that the MegaTev strongly prefers to cleave CNNNG motifs spaced 15 bp from the meganuclease-binding site, agreeing with previous studies on spacing of the cleavage motif (27). We also found that the I-TevI nuclease domain is tolerant of multiple substitutions within the CNNNG motif, with many variants cleaved better than the wild-type AAC triplet, but in general preferring A/T-rich sequences. Defining a strict consensus sequence within the motif is complicated by the observation of different levels of activity for each of the 64-possible CNNNG variants.

### Testing for off-target cleavage

Off-target cleavage is a significant concern for genome-editing endonucleases. To assess off-target cleavage of the MegaTev architecture, we took advantage of previously predicted off-targets for the I-OnuI E2 variant (the backbone meganuclease for our MegaTevs) (43). The I-OnuI E2 vari-

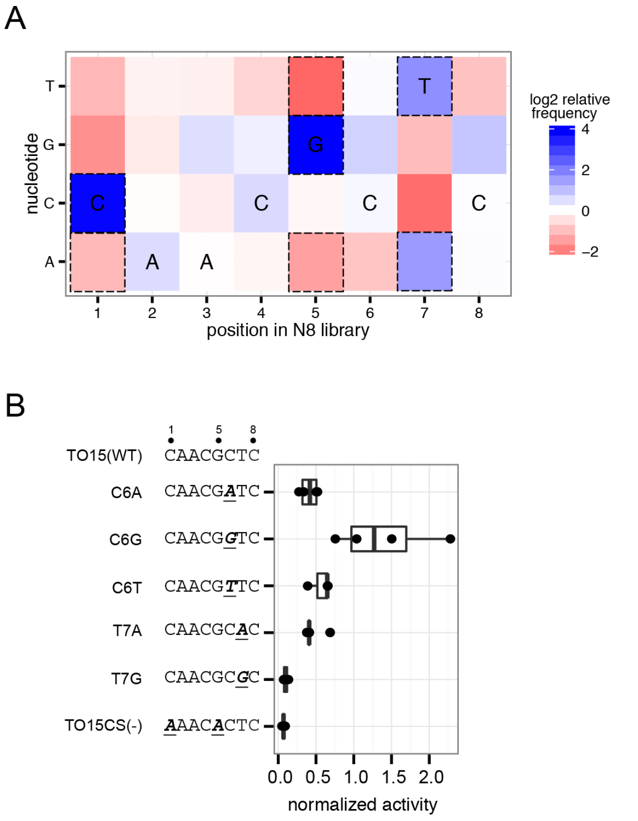

**Figure 6.** Nucleotide preference within the N8 randomized library. (**A**) Heat map for nucleotide enrichment after three rounds of selection with the Tev169-xOnu fusion. Dashed boxes represent enrichment values that are greater than two standard deviations away from the mean enrichment value for all positions. The wild-type sequence is indicated at each position. (**B**) Validation of nucleotide preferences within the N8-randomized region. Boxplot of Tev169-xOnu activity on TO15 substrates with point mutants indicated by underlined and italicized bold-type font. Activity is normalized to Tev169-xOnu on the TO15 substrate. Each mutant was assayed at least three times. Sequences are displayed on the left of the graph with the mutations in bold type and underlined.

ant was optimized to cleave a site within the human *MAO-B* gene, and a CNNNG motif is positioned 11 bp upstream of the I-OnuI E2 binding site. A number of the top-ranked off-target sites also possess CNNNG motifs within 30 bp of the I-OnuI E2 binding site (Figure 7A), suggesting that they might be substrates for the I-TevI nuclease domain. These sites differ in the spacing of the CNNNG motif from the I-OnuI E2 binding site, and also in the NNN triplet of the cleavage motif. We tested for cleavage at these sites using T7EI digestion of PCR products amplified from HEK 293 cells that were transfected with the dual active Tev169-Onu MegaTev, and by deep sequencing of the PCR products (Table 1). As shown in Figure 7B and Table 1, we observed very low indel levels at the three off-target sites. We attribute undetectable (or extremely low) activity at these sites to the sub-optimal spacing of the CNNNG motif from the I-OnuI site, and to the presence of NNN triplets that are weakly active as judged by the yeast DNA repair assay. While the sites tested represent a small number of the potential off-target sites, our data suggest that very low levels of off-target cleavage will be observed with the MegaTev at off-target sites where the CNNNG motif is not optimally spaced or where the NNN central triplet does not support robust activity.

## DISCUSSION

A number of characteristics of the I-TevI nuclease domain and meganucleases make them well suited for genome-editing applications. Of relevance to the current study is the fact that cleavage by each enzyme generates different length non-cohesive overhangs (2-nt 3′ overhangs for I-TevI and 4-nt 3′ overhangs for meganucleases) (47–49). I-TevI remains bound to its cleavage products and protects DNA ends from exonucleolytic resection, affecting the extent and directionality of DNA repair events (50). Kinetic studies have also shown that product release by meganucleases is rate limiting (51), and denaturation of meganuclease–DNA complexes is often required to resolve cleavage products in agarose gels (52). Our sequencing data largely support this idea, as many reads display no evidence of exonucleolytic processing up- or downstream of the I-TevI or I-OnuI E2 cleavage sites. End-sequestration by the single-chain MegaTev would also minimize DNA rearrangements such as translocations, inversions or duplications, as is observed with simultaneous expression of multiple genome-editing nucleases in multiplexing experiments. It is noteworthy that the fragments excised by the dual active MegaTev are similar in length to repair intermediates in the human nucleotide excision repair pathway (53). Thus, the MegaTevs may also be useful as regents for the DNA repair field, particularly if MegaTev nicking variants that excise only a single strand can be developed.

The MegaTevs used in this study were targeted to model DNA substrates. To be generally useful, the MegaTev platform must be able to target a range of sequences. Engineering meganuclease specificity has been greatly accelerated by a detailed understanding of protein–DNA contacts through crystallographic, computational and biochemical studies, and by improvements in screening methodologies (43,54–57). Recent efforts suggest that 1 in 300 bp can be targeted by the current set of meganucleases for which detailed protein–DNA contact maps are available (58). The targeting range of the MegaTev platform can also be increased by fusing the I-TevI domain to different meganucleases, as shown with the I-LtrI fusions. An alternative approach to generating MegaTevs that can target sites with a variety of nucleotide compositions could be achieved by fusing different GIY-YIG nuclease domains with distinct cleavage preferences to the same meganuclease backbone. Our preliminary studies in this regard have generated active enzymes by fusion of the GIY-YIG domain from the I-TuIa homing endonuclease to I-OnuI (Wolfs,J.M. and Edgell,D.R., unpublished results) (59).

Precise targeting and prediction of off-target sites will also require a detailed understanding of the nucleotide requirements of the GIY-YIG nuclease domain. In the case of I-TevI, past studies revealed that the nuclease domain required a 5′-CNNNG-3′ cleavage motif (45,46) and that the linker was tolerant of multiple substitutions within the DNA spacer that separates the cleavage motif from binding site (44). More recently, the I-TevI cleavage motif was defined as CDDHGS ($D$ = A,G,T; $H$ = A,C,T; $S$ = G

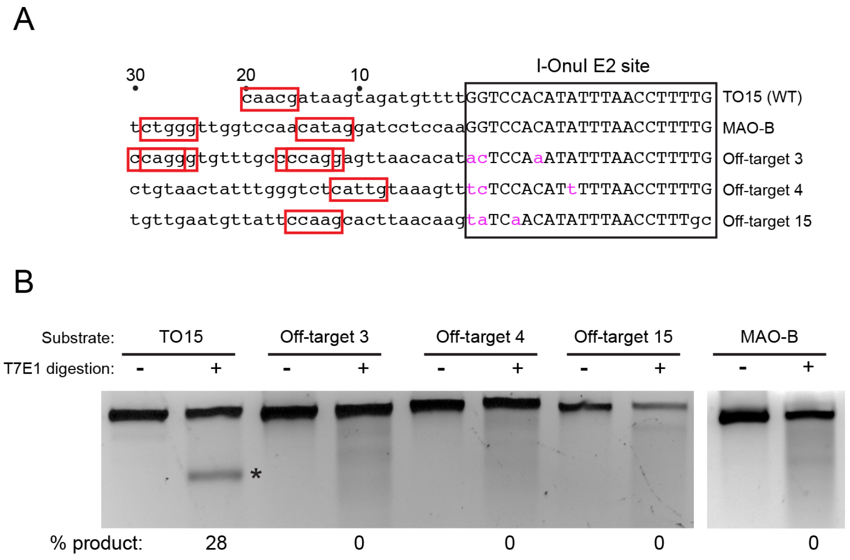

**Figure 7.** Surveyor assay for off-target cleavage. (**A**) Schematic showing the TO15, MAO-B and three off-target sites. The I-OnuI E2 site is boxed, with the nucleotide differences in pink lower case font, while the upstream sequences are in lower case black font. Numbering is relative to the 5′ end of the I-OnuI binding site. Potential CNNNG cleavage motifs are highlighted in red boxes. (**B**) T7 endonuclease I (T7EI) digests of PCR products corresponding to the indicated target sites amplified from HEK 293 cells transfected the dual active site MegaTev. The extent of T7EI cleavage is indicated below each lane.

or C) in the context of a monomeric TALEN architecture (cTALENs) (28). This consensus sequence differs from that observed for the Tev-mTALEN architecture (60) and for the observed nucleotide preferences of the MegaTevs. One reason may be that the cTALEN study examined cleavage preference within a CNNNG motif positioned 7 bp from the TALE binding site, as we find this distance is not permissive for efficient cleavage in either the MegaTev or Tev-mTALEN architecture. Our screen of DNA spacer length variation, in contrast, shows a clear spacing preference of 15 bp with MegaTevs derived from both I-OnuI and I-LtrI (Figure 1B). Our results also revealed a wide range of tolerance to substitution within the CNNNG motif, with some NNN triplets cleaved more efficiently than the wild-type AAC sequence. We anticipate that both the spacing and sequence requirement of the CNNNG motif will 'de-toxify' off-target cleavage, for the simple reason that not all off-target sites will have a permissive motif positioned appropriately from the meganuclease binding site. Indeed, our data, while representing a small number of potential off-target sites, revealed no detectable cleavage by T7EI assays, and an indel rate not significantly different than that observed for mock-transfected cells as judged by deep sequencing.

In summary, the MegaTevs represent a novel fusion of two different active sites to generate a dual nuclease with a high efficiency of gene disruption without the need to over-express DNA end-processing enzymes. The compact size of the MegaTev, coupled with the high fidelity imparted by the specificity of the I-TevI and meganuclease domains, makes it suitable for genome-engineering applications where minimizing off-target effects is paramount.

## SUPPLEMENTARY DATA

Supplementary Data are available at NAR Online.

## ACKNOWLEDGMENTS

We thank David Carter at the London Regional Genomics Facility and Greg Gloor for discussion regarding next-generation sequence, and Benjamin Kleinstiver for discussion regarding design and testing of the nucleases.

## FUNDING

Natural Sciences and Engineering Research Council of Canada (NSERC) Discovery Grant [to D.R.E.]; Canadian Institutes of Health Research (CIHR) Operating Grant [MOP 114958 to C.S.-P.]; NSERC PGS-D Scholarship [to J.M.W.]. Funding for open access charge: NSERC Discovery Grant [to D.R.E.].
*Conflict of interest statement.* None declared.

## References

1. Gaj,T., Gersbach,C.A. and Barbas,C.F. III (2013) ZFN, TALEN, and CRISPR/Cas-based methods for genome engineering. *Trends Biotechnol.*, **31**, 397–405.
2. Scharenberg,A.M., Duchateau,P. and Smith,J. (2013) Genome engineering with TAL-effector nucleases and alternative modular nuclease technologies. *Curr. Gene Ther.*, **13**, 291–303.
3. Christian,M., Cermak,T., Doyle,E.L., Schmidt,C., Zhang,F., Hummel,A., Bogdanove,A.J. and Voytas,D.F. (2010) Targeting DNA double-strand breaks with TAL effector nucleases. *Genetics*, **186**, 757–761.
4. Li,T., Huang,S., Zhao,X., Wright,D.A., Carpenter,S., Spalding,M.H., Weeks,D.P. and Yang,B. (2011) Modularly assembled designer TAL effector nucleases for targeted gene knockout and gene replacement in eukaryotes. *Nucleic Acids Res.*, **39**, 6315–6325.
5. Kim,Y.G., Shi,Y., Berg,J.M. and Chandrasegaran,S. (1997) Site-specific cleavage of DNA-RNA hybrids by zinc finger/FokI cleavage domain fusions. *Gene*, **203**, 43–49.
6. Sander,J.D., Dahlborg,E.J., Goodwin,M.J., Cade,L., Zhang,F., Cifuentes,D., Curtin,S.J., Blackburn,J.S., Thibodeau-Beganny,S., Qi,Y. *et al.* (2011) Selection-free zinc-finger-nuclease engineering by context-dependent assembly (CoDA). *Nat. Methods*, **8**, 67–69.

7. Bitinaite,J., Wah,D.A., Aggarwal,A.K. and Schildkraut,I. (1998) FokI dimerization is required for DNA cleavage. *Proc. Natl. Acad. Sci. U.S.A.*, **95**, 10570–10575.
8. Vanamee,E.S., Santagata,S. and Aggarwal,A.K. (2001) FokI requires two specific DNA sites for cleavage. *J. Mol. Biol.*, **309**, 69–78.
9. Smith,J., Bibikova,M., Whitby,F.G., Reddy,A.R., Chandrasegaran,S. and Carroll,D. (2000) Requirements for double-strand cleavage by chimeric restriction enzymes with zinc finger DNA-recognition domains. *Nucleic Acids Res.*, **28**, 3361–3369.
10. Gupta,A., Meng,X., Zhu,L.J., Lawson,N.D. and Wolfe,S.A. (2011) Zinc finger protein-dependent and -independent contributions to the in vivo off-target activity of zinc finger nucleases. *Nucleic Acids Res.*, **39**, 381–392.
11. Pattanayak,V., Ramirez,C.L., Joung,J.K. and Liu,D.R. (2011) Revealing off-target cleavage specificities of zinc-finger nucleases by in vitro selection. *Nat. Methods*, **8**, 765–770.
12. Gabriel,R., Lombardo,A., Arens,A., Miller,J.C., Genovese,P., Kaeppel,C., Nowrouzi,A., Bartholomae,C.C., Wang,J., Friedman,G. *et al.* (2011) An unbiased genome-wide analysis of zinc-finger nuclease specificity. *Nat. Biotechnol.*, **29**, 816–823.
13. Burgess,D.J. (2013) Technology: a CRISPR genome-editing tool. *Nat. Rev. Genet.*, **14**, 80.
14. Pennisi,E. (2013) The CRISPR craze. *Science*, **341**, 833–836.
15. Nishimasu,H., Ran,F.A., Hsu,P.D., Konermann,S., Shehata,S.I., Dohmae,N., Ishitani,R., Zhang,F. and Nureki,O. (2014) Crystal structure of Cas9 in complex with guide RNA and target DNA. *Cell*, **156**, 935–949.
16. Jinek,M., Chylinski,K., Fonfara,I., Hauer,M., Doudna,J.A. and Charpentier,E. (2012) A programmable dual-RNA-guided DNA endonuclease in adaptive bacterial immunity. *Science*, **337**, 816–821.
17. Sternberg,S.H., Redding,S., Jinek,M., Greene,E.C. and Doudna,J.A. (2014) DNA interrogation by the CRISPR RNA-guided endonuclease Cas9. *Nature*, **507**, 62–67 .
18. Jinek,M., Jiang,F., Taylor,D.W., Sternberg,S.H., Kaya,E., Ma,E., Anders,C., Hauer,M., Zhou,K., Lin,S. *et al.* (2014) Structures of Cas9 endonucleases reveal RNA-mediated conformational activation. *Science*,**343**, 124.
19. Stoddard,B.L. (2011) Homing endonucleases: from microbial genetic invaders to reagents for targeted DNA modification. *Structure*, **19**, 7–15.
20. Silva,G., Poirot,L., Galetto,R., Smith,J., Montoya,G., Duchateau,P. and Paques,F. (2011) Meganucleases and other tools for targeted genome engineering: perspectives and challenges for gene therapy. *Curr. Gene Ther.*, **11**, 11–27.
21. Gaj,T., Mercer,A.C., Sirk,S.J., Smith,H.L. and Barbas,C.F. III (2013) A comprehensive approach to zinc-finger recombinase customization enables genomic targeting in human cells. *Nucleic Acids Res.*, **41**, 3937–3946.
22. Gordley,R.M., Gersbach,C.A. and Barbas,C.F. III (2009) Synthesis of programmable integrases. *Proc. Natl. Acad. Sci. U.S.A.*, **106**, 5053–5058.
23. Fonfara,I., Curth,U., Pingoud,A. and Wende,W. (2011) Creating highly specific nucleases by fusion of active restriction endonucleases and catalytically inactive homing endonucleases. *Nucleic Acids Res.*, **40**, 847–860.
24. Schierling,B., Dannemann,N., Gabsalilow,L., Wende,W., Cathomen,T. and Pingoud,A. (2011) A novel zinc-finger nuclease platform with a sequence-specific cleavage module. *Nucleic Acids Res.*, **40**, 2623–2638.
25. Yanik,M., Alzubi,J., Lahaye,T., Cathomen,T., Pingoud,A. and Wende,W. (2013) TALE-PvuII fusion proteins—novel tools for gene targeting. *PLoS ONE*, **8**, e82539.
26. Boissel,S., Jarjour,J., Astrakhan,A., Adey,A., Gouble,A., Duchateau,P., Shendure,J., Stoddard,B.L., Certo,M.T., Baker,D. *et al.* (2013) megaTALs: a rare-cleaving nuclease architecture for therapeutic genome engineering. *Nucleic Acids Res.*, **42**, 2591–2601.
27. Kleinstiver,B.P., Wolfs,J.M., Kolaczyk,T., Roberts,A.K., Hu,S.X. and Edgell,D.R. (2012) Monomeric site-specific nucleases for genome editing. *Proc. Natl. Acad. Sci. U.S.A.*, **109**, 8061–8066.
28. Beurdeley,M., Bietz,F., Li,J., Thomas,S., Stoddard,T., Juillerat,A., Zhang,F., Voytas,D.F., Duchateau,P. and Silva,G.H. (2013) Compact designer TALENs for efficient genome engineering. *Nat. Commun.*, **4**, 1762.
29. Lieber,M.R. (2011) The mechanism of double-strand DNA break repair by the nonhomologous DNA end-joining pathway. *Annu. Rev. Biochem.*, **79**, 181–211.
30. McVey,M. and Lee,S.E. (2008) MMEJ repair of double-strand breaks (director's cut): deleted sequences and alternative endings. *Trends Genet.*, **24**, 529–538.
31. Shrivastav,M., De Haro,L.P. and Nickoloff,J.A. (2008) Regulation of DNA double-strand break repair pathway choice. *Cell Res.*, **18**, 134–147.
32. Certo,M.T., Gwiazda,K.S., Kuhar,R., Sather,B., Curinga,G., Mandt,T., Brault,M., Lambert,A.R., Baxter,S.K., Jacoby,K. *et al.* (2012) Coupling endonucleases with DNA end-processing enzymes to drive gene disruption. *Nat. Methods*, **9**, 973–975.
33. Delacote,F., Perez,C., Guyot,V., Duhamel,M., Rochon,C., Ollivier,N., Macmaster,R., Silva,G.H., Paques,F., Daboussi,F. *et al.* (2013) High frequency targeted mutagenesis using engineered endonucleases and DNA-end processing enzymes. *PLoS ONE*, **8**, e53217.
34. Sollu,C., Pars,K., Cornu,T.I., Thibodeau-Beganny,S., Maeder,M.L., Joung,J.K., Heilbronn,R. and Cathomen,T. (2010) Autonomous zinc-finger nuclease pairs for targeted chromosomal deletion. *Nucleic Acids Res.*, **38**, 8269–8276.
35. Ran,F.A., Hsu,P.D., Lin,C.Y., Gootenberg,J.S., Konermann,S., Trevino,A.E., Scott,D.A., Inoue,A., Matoba,S., Zhang,Y. *et al.* (2013) Double nicking by RNA-guided CRISPR Cas9 for enhanced genome editing specificity. *Cell*, **154**, 1380–1389.
36. Cong,L., Ran,F.A., Cox,D., Lin,S., Barretto,R., Habib,N., Hsu,P.D., Wu,X., Jiang,W., Marraffini,L.A. *et al.* (2013) Multiplex genome engineering using CRISPR/Cas systems. *Science*, **339**, 819–823.
37. Kleinstiver,B.P., Fernandes,A., Gloor,G.B. and Edgell,D.R. (2010) A unified genetic, computational, and experimental framework identifies noon-conserved residues as critical for function of the homing endonuclease I-BmoI. *Nucleic Acids Res.*, **38**, 2411–2427.
38. Townsend,J.A., Wright,D.A., Winfrey,R.J., Fu,F., Maeder,M.L., Joung,J.K. and Voytas,D.F. (2009) High-frequency modification of plant genes using engineered zinc-finger nucleases. *Nature*, **459**, 442–445.
39. Edgell,D.R., Stanger,M.J. and Belfort,M. (2003) Importance of a single base pair for discrimination between intron-containing and intronless alleles by endonuclease I-*Bmo*I. *Curr. Biol.*, **13**, 973–978.
40. Wickham,H. (2009) *ggplot2: Elegant Graphics for Data Analysis*. Springer, New York, NY.
41. R Core Team (2013) *R: A language and environment for statistical computing*. R Foundation for Statistical Computing. Vienna, Austria.
42. Wolfs,J.M., Kleinstiver,B.P. and Edgell,D.R. (2014) Rapid screening of endonuclease target site preference using a modified bacterial two-plasmid selection. *Methods Mol. Biol.*, **1123**, 97–104.
43. Takeuchi,R., Lambert,A.R., Mak,A.N., Jacoby,K., Dickson,R.J., Gloor,G.B., Scharenberg,A.M., Edgell,D.R. and Stoddard,B.L. (2011) Tapping natural reservoirs of homing endonucleases for targeted gene modification. *Proc. Natl. Acad. Sci. U.S.A.*, **108**, 13077–13082.
44. Bryk,M., Quirk,S.M., Mueller,J.E., Loizos,N., Lawrence,C. and Belfort,M. (1993) The *td* intron endonuclease I-*Tev*I makes extensive sequence-tolerant contacts across the minor groove of its DNA target. *EMBO J.*, **12**, 2141–2149.
45. Edgell,D.R., Stanger,M.J. and Belfort,M. (2004) Coincidence of cleavage sites of intron endonuclease I-TevI and critical sequences of the host thymidylate synthase gene. *J. Mol. Biol.*, **343**, 1231–1241.
46. Bryk,M., Belisle,M., Mueller,J.E. and Belfort,M. (1995) Selection of a remote cleavage site by I-*Tev*I, the *td* intron-encoded endonuclease. *J. Mol. Biol.*, **247**, 197–210.
47. Bell-Pedersen,D., Quirk,S., Clyman,J. and Belfort,M. (1990) Intron mobility in phage T4 is dependent upon a distinctive class of endonucleases and independent of DNA sequences encoding the intron core: mechanistic and evolutionary implications. *Nucleic Acids Res.*, **18**, 3763–3770.
48. Gimble,F.S. and Thorner,J. (1992) Homing of a DNA endonuclease gene by meiotic gene conversion in Saccharomyces cerevisiae. *Nature*, **357**, 301–306.
49. Stoddard,B.L. (2005) Homing endonuclease structure and function. *Q. Rev. Biophys.*, **38**, 49–95.

50. Mueller,J.E., Smith,D. and Belfort,M. (1996) Exon coconversion biases accompanying intron homing: battle of the nucleases. *Genes Dev.*, **10**, 2158–2166.
51. Chevalier,B., Sussman,D., Otis,C., Noel,A.J., Turmel,M., Lemieux,C., Stephens,K., Monnat,R.J. Jr and Stoddard,B.L. (2004) Metal-dependent DNA cleavage mechanism of the I-CreI LAGLIDADG homing endonuclease. *Biochemistry*, **43**, 14015–14026.
52. Argast,G.M., Stephens,K.M., Emond,M.J. and Monnat,R.J. Jr (1998) I-*Ppo*I and I-*Cre*I homing site sequence degeneracy determined by random mutagenesis and sequential in vitro enrichment. *J. Mol. Biol.*, **280**, 345–353.
53. Fagbemi,A.F., Orelli,B. and Scharer,O.D. (2011) Regulation of endonuclease activity in human nucleotide excision repair. *DNA Repair (Amst)*, **10**, 722–729.
54. Jarjour,J., West-Foyle,H., Certo,M.T., Hubert,C.G., Doyle,L., Getz,M.M., Stoddard,B.L. and Scharenberg,A.M. (2009) High-resolution profiling of homing endonuclease binding and catalytic specificity using yeast surface display. *Nucleic Acids Res.*, **37**, 6871–6880.
55. Thyme,S.B., Jarjour,J., Takeuchi,R., Havranek,J.J., Ashworth,J., Scharenberg,A.M., Stoddard,B.L. and Baker,D. (2009) Exploitation of binding energy for catalysis and design. *Nature*, **461**, 1300–1304.
56. Arnould,S., Chames,P., Perez,C., Lacroix,E., Duclert,A., Epinat,J.C., Stricher,F., Petit,A.S., Patin,A., Guillier,S. *et al.* (2006) Engineering of large numbers of highly specific homing endonucleases that induce recombination on novel DNA targets. *J. Mol. Biol.*, **355**, 443–458.
57. Smith,J., Grizot,S., Arnould,S., Duclert,A., Epinat,J.C., Chames,P., Prieto,J., Redondo,P., Blanco,F.J., Bravo,J. *et al.* (2006) A combinatorial approach to create artificial homing endonucleases cleaving chosen sequences. *Nucleic Acids Res.*, **34**, e149.
58. Takeuchi,R., Choi,M. and Stoddard,B.L. (2014) Redesign of extensive protein-DNA interfaces of meganucleases using iterative cycles of in vitro compartmentalization. *Proc. Natl. Acad. Sci. U.S.A.*, **111**, 4061–4066.
59. Sandegren,L. and Sjöberg,B.-M. (2004) Distribution, sequence homology, and homing of group I introns among T-even-like bacteriophages: evidence for recent transfer of old introns. *J. Biol. Chem.*, **279**, 22218–22227.
60. Kleinstiver,B.P., Wang,L., Wolfs,J.A., Kolaczyk,T., McDowell,B., Wang,X., Schild-Poulter,C., Bogdanove,A.J. and Edgell,D.R. (2014) The I-TevI nuclease and linker domains contribute to the specificity of monomeric TALENs. *G3 (Bethesda)*, **4**, 1155–1165.
